# Artificial Intelligence (AI) in Saxitoxin Research: The Next Frontier for Understanding Marine Dinoflagellate Toxin Biosynthesis and Evolution

**DOI:** 10.3390/toxins18010026

**Published:** 2026-01-05

**Authors:** Buhari Lawan Muhammad, Han-Sol Kim, Ibrahim Aliyu, Harisu Abdullahi Shehu, Jang-Seu Ki

**Affiliations:** 1 Department of Life Science, Sangmyung University, Seoul 03016, Republic of Korea; 2 Institute of Natural Science, Sangmyung University, Seoul 03016, Republic of Korea; 3 Department of Intelligent Electronics and Computer Engineering, Chonnam National University, Gwangju 61186, Republic of Korea; 4 School of Engineering and Computer Science, Victoria University of Wellington, 6140 Wellington, New Zealand; harisushehu@ecs.vuw.ac.nz

**Keywords:** machine learning integration, multi-omics analysis, deep learning, paralytic shellfish poisoning (PSP), harmful algal blooms, saxitoxin, dinoflagellates

## Abstract

Saxitoxin (STX) is one of the most potent marine neurotoxins, produced by several species of freshwater cyanobacteria and marine dinoflagellates. Although omics-based approaches have advanced our understanding of STX biosynthesis in recent decades, the origin, regulation, and ecological drivers of STX in dinoflagellates remain poorly resolved. Specifically, dinoflagellate STX biosynthetic genes (*sxt*) are extremely fragmented, inconsistently expressed, and unevenly distributed between toxic and non-toxic taxa. Environmental studies further report inconsistent relationships between abiotic factors and STX production, suggesting regulation across multiple genomic, transcriptional, post-transcriptional, and epigenetic levels. These gaps prevent a comprehensive understanding of STX biosynthesis in dinoflagellates and limit the development of accurate predictive models for harmful algal blooms (HABs) and paralytic shellfish poisoning (PSP). Artificial intelligence (AI), including machine learning and deep learning, offers new opportunities in ecological pattern recognition, molecular annotation, and data-driven prediction. This review explores the current state of knowledge and persistent knowledge gaps in dinoflagellate STX research and proposes an AI-integrated multi-omics framework highlighting recommended models for *sxt* gene identification (e.g., DeepFRI, ProtTrans, ESM-2), evolutionary reconstruction (e.g., PhyloGAN, GNN, PhyloVAE, NeuralNJ), molecular regulation (e.g., MOFA+, LSTM, GRU, DeepMF), and toxin prediction (e.g., XGBoost, LightGBM, LSTM, ConvLSTM). By integrating AI with diverse biological datasets, this novel framework outlines how AI can advance fundamental understanding of STX biosynthesis and inform future applications in HAB monitoring, seafood safety, and PSP risk management in aquaculture and fisheries.

## 1. Introduction

The UN Decade of Ocean Science for Sustainable Development (2021–2030) calls for innovative and timely scientific approaches to ensure a safe and sustainable ocean, one where human health, ecosystems, and economies coexist in balance [1]. Yet, despite significant progress in marine research, the ocean continues to present natural hazards with profound biological and socioeconomic consequences [2]. Among these, marine biotoxins remain a persistent and complex threat to food security, coastal livelihoods, and global public health [3,4].

Saxitoxins (STXs) are among the most potent natural biotoxins known, produced primarily by certain marine dinoflagellates and freshwater cyanobacteria [5,6,7,8]. These toxins are the primary cause of paralytic shellfish poisoning (PSP), a severe neurological disorder resulting from the consumption of contaminated seafood [8,9]. Globally, approximately 2000 cases of PSP are reported annually, with mortality rates reaching up to 15%, underscoring the continuing public health and economic burden associated with harmful algal blooms (HABs) [10,11,12,13,14].

The growing incidence of HAB-related poisonings is increasingly linked to climate change, intensifying the urgency to investigate toxin-producing species [2,15,16]. Rising temperatures and shifting ocean conditions are expanding the range and frequency of HAB events worldwide [2]. Evidence indicates that climate-driven alterations in temperature, salinity, and nutrient dynamics shape the growth and toxicity of PSP-producing taxa such as the marine dinoflagellate *Alexandrium* [17,18,19,20,21]. Early warnings on toxicity would require knowing the biochemical processes responsible for the synthesis of STX-related compounds in dinoflagellates in response to environmental changes [5,6]. Given these emerging risks, a comprehensive understanding of STX biosynthesis, regulation, and distribution remains critical for ensuring both ocean and public health security.

Despite decades of research, significant gaps remain in understanding the molecular and ecological mechanisms underlying STX biosynthesis in dinoflagellates. Although extensive genetic and transcriptomic data are now available [22], their complexity continues to obscure the evolutionary dynamics of saxitoxin biosynthesis genes (*sxt*), patterns of gene expression, and climate-driven influences on toxin production, a paradox where each discovery seems to reveal even deeper layers of complexity. These challenges emphasize the urgent need for innovative computational and integrative approaches to unravel the complex nature of STX biosynthesis.

In this sense, machine learning (ML) and artificial intelligence (AI) have become powerful tools, providing new capabilities in ecological pattern recognition, molecular annotation, and data-driven prediction [23,24,25]. AI has the potential to completely change how we identify, model, and comprehend saxitoxin-producing organisms by combining genomics, transcriptomics, proteomics, metabolomics, and environmental datasets [25,26,27,28,29,30]. This review examines the current state and persistent knowledge gaps in dinoflagellate saxitoxin research and proposes the integration of multi-omics data and AI to advance the field. Furthermore, it introduces an innovative research framework that applies AI to *sxt* gene identification, evolutionary inference, molecular regulation, and toxin prediction. With this integration, saxitoxin research is moving from decades of contradictory results into a new era of comprehensive understanding of dinoflagellate toxins. The AI integration bridges the gap between basic science and practical applications in marine toxin management by unifying fragmented data, elucidating biological mechanisms, and turning knowledge into action, positioning these advancements within the broader context of ocean health and public health security.

## 2. Saxitoxin Biosynthesis: Cyanobacteria vs. Dinoflagellates

The biosynthesis of saxitoxins was first elucidated in freshwater cyanobacteria (*Raphidiopsis brookii*, *Anabaena circinalis*, *Aphanizomenon* sp., *Cylindrospermopsis raciborskii*, and *Microseira wollei*), where a well-characterized *sxt* gene cluster comprising approximately 30 genes (*sxtA*-*sxtZ*) orchestrates modular enzymatic steps [8,20,31,32]. These include core genes *(sxtA*, *sxtB*, *sxtD*, *sxtG*, *sxtH*/*T*, *sxtI*, *sxtS*, and *sxtU*) and various tailoring and transporting enzymes responsible for the structural diversity of STX and its derivatives, such as GTX2, GTX3, and neosaxitoxin (NeoSTX) [32,33]. Cyanobacterial STX biosynthesis serves as a foundational model for understanding the genetic and enzymatic logic of saxitoxin production [8,34].

In contrast, dinoflagellates exhibit a highly fragmented and complex distribution of *sxt* homologs and produce a broader diversity of STX derivatives, including sulfated forms such as C1/C2, GTX4, and GTX6, which are only rarely produced by cyanobacteria [35,36,37]. Among dinoflagellates, *Alexandrium* spp. (approximately ten toxic species), *Pyrodinium bahamense*, *Gymnodinium catenatum*, and *Centrodinium punctatum* are the only confirmed saxitoxin (STX)-producing taxa [37,38,39,40,41,42]. Core genes such as *sxtA* (particularly the *sxtA4* domain) and *sxtG* have been identified across most toxic species [36,39,42,43]. In contrast, non-toxic species such as *Alexandrium fragae*, *A. fraterculus*, *Scrippsiella trochoidea*, *Margalefidinium polykrikoides*, and freshwater *Palatinus apiculatus* typically lack these core genes [42,44,45,46]. However, the distribution of *sxt* genes does not always correspond to toxicity status. For example, *C. punctatum* reportedly lacks *sxtA4* [47], and *G. catenatum* lacks *sxtB* [39]. Moreover, complete *sxt* gene sets have been detected in non-toxic strains of *Alexandrium* spp. [37,48,49,50] and in the non-toxic *G. smaydae* [51]. These inconsistencies highlight the complex evolutionary dynamics of *sxt* homologs in dinoflagellates [52], which seem to be different from cyanobacteria [53] and raise a critical question: which specific genes and pathways ultimately determine saxitoxin biosynthesis in this group?

Furthermore, comparative transcriptomic analyses further reveal substantial variation in *sxt* gene expression across dinoflagellate species. Nevertheless, the relationship between *sxt* gene expression and actual toxin production remains inconsistent. For example, some studies reported that *sxtA4* and *sxtG* expression in *Alexandrium* species were significantly reduced in non-toxic strains [49,54,55,56]. In contrast, other studies observed no significant difference in *sxt* gene expression between toxic and non-toxic individuals, suggesting that STX biosynthesis in dinoflagellates may be regulated beyond transcription, potentially at post-transcriptional or translational levels [37,57,58].

In addition, although several abiotic stressors, such as nutrient limitation, salinity shifts, and temperature or light fluctuations, have been linked to changes in STX production [17,18,19,21,59,60], current evidence does not yet support a consistent, predictive model connecting environmental triggers, *sxt* gene expressions, and toxin yield [19,61,62,63]. This inconsistency implies that additional regulatory layers, including epigenetic modulation [64,65] or microbial community interactions [66], may play key roles in mediating STX biosynthesis [5,22]. Collectively, these observations highlight a critical knowledge gap in our understanding of how *sxt* genes, environmental factors, and toxin production interact.

## 3. Evolutionary Perspective

The inconsistent relationship between *sxt* gene expression, environmental drivers, and STX production suggests that *sxt* gene evolution in dinoflagellates involves complex mechanisms beyond simple vertical inheritance [34,36]. Early hypotheses proposed that *sxt* genes were acquired in dinoflagellates via horizontal gene transfer (HGT) from cyanobacteria to a common ancestor of *Alexandrium* and *P. bahamense*, with *G. catenatum* later acquiring them from *Alexandrium* secondarily via HGT [36]. Phylogenetic analyses appeared to support this, as the STX-producing *P. bahamense* and *C. punctatum* consistently cluster within the *Alexandrium* lineage, whereas *G. catenatum* forms a more distant branch [47,51]. However, recent studies suggest that cyanobacteria and dinoflagellates may have acquired *sxt* genes independently rather than through a single ancient HGT event [34,67]. Consistently, *G. catenatum* forms a distinct lineage in *sxt* phylogenies, contradicting the idea of secondary acquisition from *Alexandrium* [39,67].

In dinoflagellates, current evidence suggests that *sxt* genes were acquired early in dinoflagellate evolution and subsequently shaped by lineage-specific loss, duplication, and divergence [42,67]. This has produced the current patchy distribution of *sxt* homologs, in which some non-toxic dinoflagellate species retain partial or non-functional remnants of the *sxt* genes [34,36,39,45,46,50,51,67]. An alternative hypothesis is that the *sxt* gene cluster may have originated independently in multiple dinoflagellate lineages through a complex history of horizontal gene transfer (HGT), potentially involving bacterial donors such as Proteobacteria or Actinobacteria, like the recurrent HGT events observed in cyanobacteria [33].

All these interpretations remain highly speculative. The evolutionary genomic origins of saxitoxin production and the underlying *sxt* gene clusters in dinoflagellates remain an open question. Did STX biosynthesis arise once or independently multiple times in dinoflagellates? Do retained *sxt* homologs represent active enzymes or evolutionary relics? What ecological and environmental forces govern the retention, silencing, or loss of these genes across lineages? Until the evolutionary history of *sxt* genes is clearly resolved, our understanding of saxitoxin biosynthesis will remain incomplete, limiting predictive capacity and constraining efforts to anticipate or mitigate its ecological and public health impacts.

## 4. Knowledge Gaps and Challenges in Dinoflagellates

Despite remarkable advances in sequencing technologies and comparative omics, significant gaps remain in our understanding of STX biosynthesis in dinoflagellates. Five critical gaps remain in saxitoxin research:

1. Incomplete gene clusters: No dinoflagellate genome has yet revealed a complete *sxt* gene cluster due to extensive genome fragmentation, repetitive content, and multiple isoforms.
2. Regulatory complexity; Mechanisms involving transcriptional regulation, alternative splicing, and epigenetic control are poorly characterized, limiting accurate inference of toxin biosynthesis from sequence data.
3. Functional validation: Experimental confirmation of *sxt* gene function remains difficult because of the extraordinarily large, polyploid, and repetitive genomes of dinoflagellates, combined with a lack of robust genetic tools.
4. Environmental modulation: Toxin production varies under multiple interacting stressors (temperature, salinity, nutrient availability, and light), yet the molecular links between environmental cues and toxin biosynthesis remain unclear.
5. Unresolved evolutionary origins and diversification; The evolutionary history of *sxt* genes in dinoflagellates remains poorly resolved.

These uncertainties are compounded by the extraordinarily large and highly repetitive genomes of dinoflagellates, characterized by extensive gene duplication and alternative splicing [68,69,70], which present major challenges for accurately reconstructing *sxt* gene clusters and elucidating their regulatory networks [69]. Additionally, field observations add further complexity, as dinoflagellate blooms arise under multifactorial environmental conditions and are modulated by intricate ecological interactions such as microbiome composition, grazing pressure, and nutrient competition, factors that are difficult to replicate in controlled laboratory settings [17,71]. Table 1 below summarizes the major knowledge gaps in *sxt* gene cluster architecture, regulation, environmental modulation, and evolutionary origins in dinoflagellates.

Collectively, these challenges underscore why conventional approaches alone have been insufficient to fully resolve the evolutionary dynamics and functional complexity of *sxt* genes, despite decades of research [22,52]. Addressing these complexities requires analytical strategies capable of integrating heterogeneous, high-dimensional data across genomic, transcriptomic, environmental, and evolutionary scales. In this context, artificial intelligence and machine learning offer powerful tools to combine multi-omics datasets that can lay the groundwork for predictive, data-driven frameworks that not only advance our understanding of dinoflagellate toxins but also enhance risk assessment, forecasting, and early-warning capabilities in an ocean under rapid climate change [23,30,94].

## 5. Artificial Intelligence: A Future Tool in Dinoflagellate Saxitoxin Research

**Figure 1:**
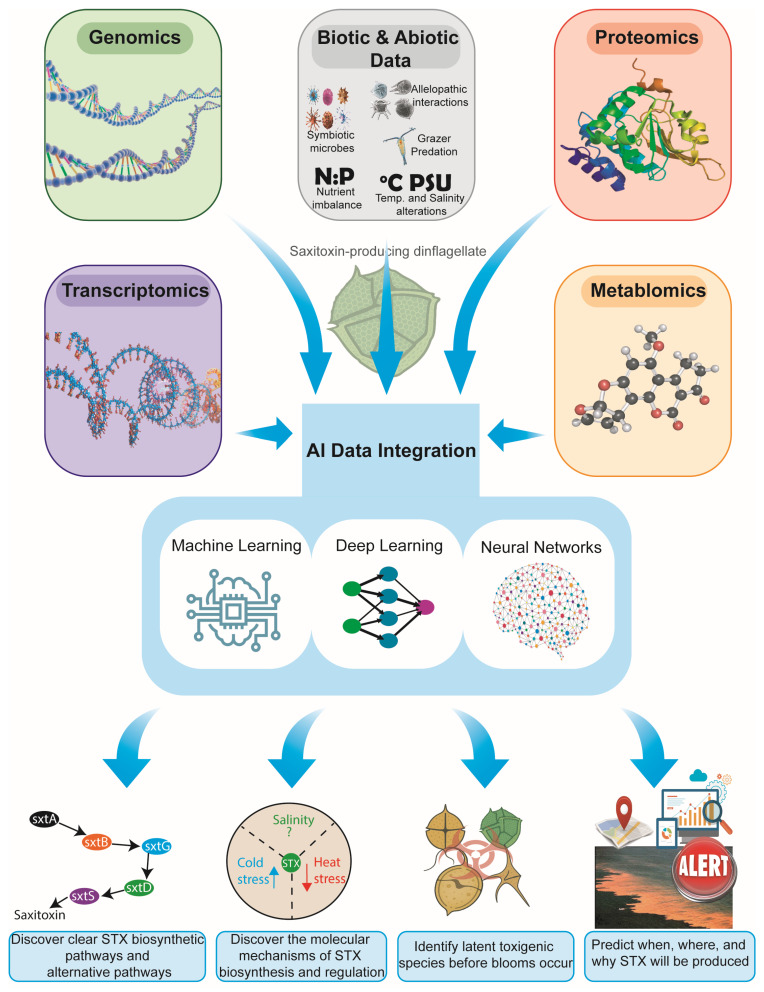
Integrated multi-omics and environmental data framework for AI-driven saxitoxin research in dinoflagellates. The figure illustrates how genomics, transcriptomics, proteomics, metabolomics, and biotic/abiotic ecological data are integrated through AI approaches, including machine learning, deep learning, and neural networks, to uncover biosynthetic pathways, elucidate molecular regulatory mechanisms, identify latent toxigenic species, and predict the timing, location, and drivers of STX production.

Artificial intelligence (AI) offers transformative opportunities for resolving long-standing challenges in saxitoxin (STX) research [30,95], particularly considering the highly complex dinoflagellate genomes, fragmented and poorly conserved *sxt* gene clusters, and the multifactorial environmental drivers that regulate toxin production. While conventional genomics and transcriptomics have provided foundational insights, AI-based approaches uniquely enable the discovery of hidden patterns, the prediction of biological outcomes, the integration of heterogeneous datasets, and the reconstruction of latent molecular structures that remain inaccessible through traditional methods [96,97,98,99]. By synthesizing multiple omics layers, including genomics, transcriptomics, proteomics, metabolomics, and environmental data, AI has the capacity to reveal previously obscured regulatory mechanisms, predict the dynamics of harmful algal blooms, and illuminate the molecular basis of saxitoxin biosynthesis with unprecedented precision (Figure 1).

Below, we review key methodological challenges and provide experimental strategies, paired with AI methods, to address them.

### 5.1. AI for Accurate Molecular Identification of sxt Genes in Dinoflagellate

Accurate identification of *sxt* genes in dinoflagellates remains one of the major challenges in saxitoxin research. Unlike cyanobacteria, where *sxt* genes occur in compact and well-defined clusters [32], dinoflagellate genomes are huge, repetitive, and rich in duplicated PKS-like sequences, obscuring gene boundaries and inflating homolog counts [52,69]. To date, several core *sxt* genes have been isolated and functionally characterized in *Alexandrium* such as *sxtA*, *sxtB*, *sxtG*, *sxtI*, and *sxtU*. Similarly, *sxt* homologs are reported across many dinoflagellate species primarily from transcriptomic data; however, in all cases these genes remain partial fragments, or expanded paralog families resulting in high false-positive rates [18,46,52,100,101]. Although definitive confirmation requires high-quality genome assemblies, full-length transcripts, and biochemical validation, such rigorous datasets are rarely available across species [5,22,52]. This limitation highlights the need for an AI-guided identification pipeline.

Deep learning–based gene prediction models, including convolutional neural networks (CNN) and transformer architectures [102,103,104,105], can detect coding signatures and functional motifs within highly repetitive or fragmented dinoflagellate sequences that traditional HMM- or BLAST-based workflows fail to distinguish [50]. Protein language models such as ESM-2, ProtT5, ProtBERT, and AlphaFold + DeepFRI generate high-dimensional embeddings that capture biochemical properties, structural constraints, and evolutionary signals [104,105,106,107] enabling functional annotation of remote homologs with limited sequence identity [104]. Specifically, DeepFRI and ProtTrans models have been shown to substantially improve Gene Ontology and enzyme function prediction accuracy in poorly annotated genomes, while maintaining robust performance under sparse training data conditions [108]. Similarly, DeepBGC has demonstrated enhanced sensitivity in detecting cryptic and atypical biosynthetic gene clusters compared with rule-based and profile-based methods, including clusters missed by antiSMASH and HMM-only pipelines [109].

These outcomes are particularly relevant to dinoflagellate *sxt* genes, which exhibit extensive fragmentation and share conserved domains with polyketide synthase (PKS) and fatty acid synthase (FAS) paralogs. Embedding-based classifiers and structural feature integration have been shown to discriminate functionally distinct enzyme families despite high sequence similarity, supporting their application for separating true *sxt* enzymes from PKS- or FAS-like homologs [110,111]. In parallel, graph neural networks (GNNs) [112] can classify complex multidomain architectures characteristic of dinoflagellate toxin genes, while ensemble pipelines integrating HMM profiles, structural predictions, and deep-learning embeddings will provide robust cross-validation [102,112]. This will make high-confidence *sxt* gene identification possible even in the absence of complete genomes or biochemical assays, transforming a previously slow, error-prone, and species-specific process into a scalable, reproducible pipeline [26,111,113]. In short, AI could shift *sxt* gene discovery from reactive validation to predictive, high-throughput discovery, fundamentally changing our ability to map toxin biosynthesis across dinoflagellate diversity. Collectively, these advances highlight a transformative opportunity to finally resolve the long-standing challenges associated with accurate *sxt* gene detection in dinoflagellates.

### 5.2. Integrative Phylogenomics and Phylogenetics of sxt Genes with AI-Enhanced Approaches for Understanding Their Evolution in Dinoflagellates

At the phylogenetic level, *sxt* genes in dinoflagellates often form clades incongruent with organismal trees, indicating recurrent horizontal acquisitions, retentions of ancestral duplicates, or differential losses across lineages [36,39,42,50]. This mosaic evolution complicates ortholog inference but also highlights the evolutionary flexibility of toxin biosynthesis pathways. Understanding the evolutionary history of *sxt* genes in dinoflagellates is essential for reconstructing the origins of STX biosynthesis and explaining the remarkable variability in toxicity across species and strains. AI-driven phylogenomic and phylogenetic approaches are proving increasingly transformative [114,115,116]. For instance, machine learning models (e.g., Random Forests, Support Vector Machines), deep learning frameworks (e.g., PhyloGAN, GNN, PhyloVAE, NeuralNJ), and reinforcement learning have been successfully applied to reconstruct phylogenies and unravel complex evolutionary processes, such as horizontal gene transfer, in other species, including bacteria [117,118].

However, in dinoflagellates, to map the evolutionary dynamics of *sxt* genes, an integrative framework is proposed, combining genome- or transcriptome-based phylogenomics with phylogenetic reconstruction of all identified *sxt* genes, augmented by AI and ML methods. However, transcriptome-based phylogenomics [119] is more practical because of the incredibly large genome of STX-producing dinoflagellates [68]. Additionally, a number of dinoflagellate species’ transcriptome data are currently accessible on public databases such as GenBank of the National Center for Biotechnology Information (NCBI). Thus, phylotranscriptomics could provide a species tree backbone using conserved single-copy genes, contextualizing gene-specific evolution [119]. AI approaches, such as embedding-based orthology inference with protein language models (ESM, ProtTrans) and GNN, can resolve fragmented or divergent transcripts, distinguishing orthologs from paralogs and improving alignments [116,120,121,122]. For *sxt* gene phylogenetics, sequences could be first curated by deep-learning classifiers (e.g., embeddings + DL-based selection), before building maximum-likelihood or Bayesian trees. Then, AI-based reconciliation [123,124] could integrate gene and species trees to detect horizontal gene transfer, gene gain/loss, and lineage-specific expansions, a strategy analogous to recent ML-driven reconciliation methods and reconciliation frameworks that model duplication, transfer, and loss events [123,124,125,126,127]. Furthermore, variational autoencoders (VAEs) and other generative deep-learning models can construct low-dimensional latent representations of protein sequence space that reflect evolutionary divergence, selective constraints, and functional relationships; such spaces have been used to model fitness landscapes and predict mutational effects among paralogs or novel variants [128,129,130,131]. Together, these AI-driven and phylogenomic approaches offer a scalable framework to resolve fragmented *sxt* gene clusters, distinguish orthologs from paralogs, infer horizontal gene transfer, duplication, and loss events, and link sequence evolution to ecological adaptation and toxin diversity, thus providing a predictive roadmap for understanding saxitoxin evolution even in the absence of complete genomes or extensive experimental validation

### 5.3. Decoding the Regulatory Mechanisms of STX Biosynthesis in Dinoflagellates Using Multi-Omics Data and an AI-Integrated Approach

Deciphering the regulation of STX biosynthesis in dinoflagellates remains a major challenge due to the complex and layered control of gene expression in these organisms [22,52]. Previous studies provide inconsistent evidence of transcriptional activity that regulates STX production [37,52,56,57]. Therefore, emerging evidence strongly suggests post-transcriptional mechanisms, including alternative transcript isoforms, RNA editing, and variations in untranslated regions (UTRs), as critical modulators of toxin production [37,64,76,132]. At present, the regulatory mechanisms of STX synthesis in dinoflagellates are not known. Additionally, omics-based studies in dinoflagellates focusing on saxitoxin biosynthesis are still sporadic and far from coming to a concrete closure [22,65,133]. This highlights the need for an integrated multi-omics (genomics, transcriptomics, proteomics, and metabolomics), e.g., [134] and an AI approach capable of simultaneously capturing transcriptional dynamics, post-transcriptional modifications, and translational output, providing a comprehensive framework to uncover the regulatory architecture of *sxt* gene expression.

**Figure 2:**
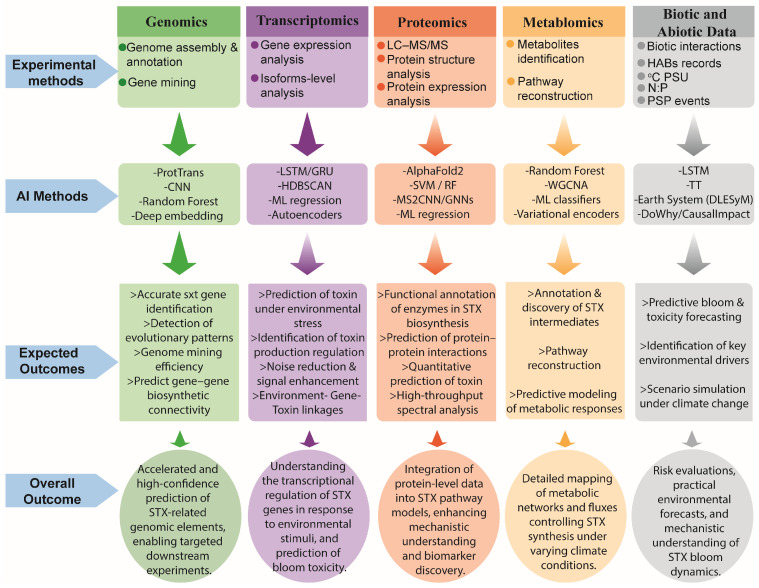
Multi-omics experimental methods, Artificial Intelligence (AI) approaches, and expected outcomes in dinoflagellate saxitoxin research. The figure summarizes how genomics, transcriptomics, proteomics, metabolomics, and biotic/abiotic environmental data are analyzed using diverse AI, machine-learning, and deep learning techniques to generate predictive, mechanistic, and functional insights. Expected outcomes include improved gene annotation, toxin-regulation modeling, enzyme function prediction, metabolic pathway reconstruction, and environmental forecasting.

Machine learning–based multi-omics integration has already proven powerful in fields such as disease diagnosis, treatment prediction, and gene regulatory network inference [27,30,99,135,136,137,138]. For example, the mechanism of salt tolerance in plants was revealed by integrating the KANMB Machine Learning Model with metabolomic and transcriptomic data [139]. These same frameworks can be leveraged for dinoflagellate saxitoxin research by combining multi-omics datasets [22,134,140]. RNA-Seq under time-course and environmental treatments could provide high-resolution profiles of transcript abundance, while ribosome profiling (Ribo-Seq) and polysome fractionation can identify which *sxt* transcripts are actively translated [134]. Furthermore, small RNA sequencing and nanopore direct RNA sequencing could reveal regulatory RNAs, RNA editing, alternative splice variants, and trans-splicing events that shape *sxt* gene expression beyond transcription; for example, SL trans-splicing is widespread in dinoflagellates [141] and nanopore DRS has been used in other eukaryotes to identify full-length isoforms, mRNA modifications, and novel splice events [142,143]. Complementing these data, AI and machine learning models can predict translational efficiency, detect dynamic regulatory patterns, and infer causal post-transcriptional networks [26,144]. Temporal deep learning models (LSTM, GRU) capture time-resolved expression dynamics, while graph-based models and Bayesian networks integrate multi-omics data to uncover hidden regulatory relationships [140]. VAEs and other integrative frameworks (e.g., MOFA+, DeepMF) can cluster regulatory states and model latent control mechanisms [145,146,147]. By combining multi-omics experiments with AI-driven analysis, it becomes possible to identify *sxt* transcripts under translational control, predict regulatory interactions, and generate scalable, predictive models of toxin biosynthesis under variable environmental conditions. Figure 2 provides an overview of the omics experimental methods, AI techniques, and expected outcomes.

### 5.4. AI-Driven Reconstruction of Saxitoxin Gene Clusters and Biosynthetic Pathways

Reconstructing the saxitoxin biosynthetic pathway in dinoflagellates remains particularly challenging because meaningful pathway inference depends on both accurate gene identification and a clear understanding of how these genes are regulated. Unlike cyanobacteria, where *sxt* genes form compact, conserved clusters with identified pathways [32,33], dinoflagellates show fragmented, dispersed, plastid-associated, and paralog-rich *sxt* architectures [34,36,48]. Moreover, increasing evidence suggests that STX biosynthesis may be species-specific, with different dinoflagellates using distinct subsets of *sxt* genes [39,51], or compensating for missing steps using unrelated PKS-like enzymes. Thus, the pathway may not be universally conserved across the group, making generalization difficult without species-level resolution.

To resolve the STX biosynthetic pathway in dinoflagellates with potential species-specific architectures, a combined experimental and computational strategy will be essential [109,148,149,150]. Long-read sequencing combined with Hi-C scaffolding has been shown to resolve complex gene neighborhoods, reveal micro-clusters, and detect lineage-specific structural rearrangements in highly repetitive genomes [151,152]. Furthermore, co-expression network approaches such as WGCNA [153], together with more advanced integrative Gene Regulatory Network (iGRN) inference methods, will help identify species-specific functional modules and regulatory circuits associated with toxin biosynthesis [136,149,153]. LC-MS/MS metabolomics will allow direct comparison of saxitoxin intermediates to determine differences in pathway completeness, while stable isotope labeling can validate enzyme functions and pathway flux in a species-dependent context [65,154]. Protein–protein interaction assays (Y2H, co-IP, AP-MS) will further uncover enzyme complexes that may differ among lineages. AI-driven methods further enhance cluster reconstruction: Graph Neural Networks (GNNs) can infer hidden biosynthetic modules from fragmented scaffolds [120], while deep-learning metabolomics frameworks (DeepMET, DeepMass) associate metabolites with candidate genes to resolve missing steps in species-specific pathways [155,156]. Finally, multi-omics integrative tools such as DeepPath [157] and NICEpath [158] can then model alternative biosynthetic routes, enabling prediction of lineage-specific *sxt* gene cluster organization and pathway variants across dinoflagellates. Together, this framework could enable the first realistic reconstruction of *sxt* gene clusters, whether canonical, fragmented, or species-specific, across diverse toxic dinoflagellates.

### 5.5. Predicting Toxicity in a Changing Ocean: AI Solutions to Understanding Saxitoxin Environmental Drivers

In the context of rapid climate change, the frequency, intensity, and geographic distribution of saxitoxin-producing harmful algal blooms (HABs) and paralytic shellfish poisoning (PSP) events are expanding into new areas, posing increasing risks to human health, fisheries, and marine ecosystems [159,160,161,162]. As HABs often arise under multifactorial environmental conditions, recent advances across aquatic toxin research, including cyanobacteria and dinoflagellate toxins, employ AI integration of biological and environmental data to understand the dynamics of HABs and forecast HABs, identify key environmental triggers, and predict bloom toxicity with high accuracy (e.g., 84%) [96,98,163,164,165,166]. In dinoflagellates, saxitoxin biosynthesis regulation is indeed a complex process, controlled by several environmental cues that are yet to be clearly defined [5,22]. Based on these, we suggest that machine learning (ML) and artificial intelligence (AI) based frameworks are well-suited for the integration and analysis of biological and environmental data from naturally occurring HABs, and that such an approach would greatly improve our ability to understand saxitoxin and predict HABs under a changing climate [167,168]. For example, deep learning models that capture interactions among environmental variables have improved riverine HAB prediction [169], while multi-horizon architectures enable robust long-term bloom forecasting by resolving complex temporal dependencies [170]. At broader spatial scales, the integration of remote sensing with AI has enhanced early detection and prediction of inland-water HABs [167], and machine-learning approaches have successfully linked environmental variability to blooms of toxin-producing taxa such as *Pseudo-nitzschia* in coastal systems [168]. Collectively, these advances demonstrate how AI can support predictive frameworks relevant to the monitoring and management of toxin-producing algal blooms.

In dinoflagellate, the effective AI-driven prediction of saxitoxin risk must be grounded in experimental and field-based observations that capture the biochemical and ecological controls of toxin production. Controlled laboratory experiments manipulating nutrients, temperature, pH, or metal concentrations provide mechanistic insight into how environmental stressors influence saxitoxin (STX) production [18,60,62,77], while co-culture and microbiome studies reveal the role of biological interactions, such as competition and symbiosis [66]. High-frequency field sampling of natural blooms, combined with environmental and community metadata, including nutrient levels, light, hydrodynamics, and microbiome composition, could enable in situ assessment of STX variability [17,96,171]. Quantitative toxin measurements (LC–MS/MS) alongside transcriptomic and metabolomic profiling could link both abiotic and biotic factors to molecular and biochemical responses [134,154]. In addition, extensive datasets on species-specific toxin analog profiles and their geographic distributions, derived from long-term monitoring programs and published toxin surveys, provide critical phenotypic labels that can be integrated with molecular and environmental data for AI model training, validation, and cross-regional generalization [172,173]. Artificial intelligence and machine-learning approaches can then integrate these heterogeneous datasets to predict toxin production and identify key drivers. Algorithms such as Random Forests, gradient boosting models (XGBoost, LightGBM), and Support Vector Regression can model *sxt* responses to both abiotic and biotic variables [174], while spatiotemporal neural networks, including LSTMs, ConvLSTMs, temporal transformers, and Earth system AI models like neural operators, would enable bloom-scale forecasting under dynamic environmental and ecological conditions [96,175,176]. Causal inference frameworks, such as DoWhy [177] and CausalImpact [178], allow researchers to distinguish correlation from causation, revealing which environmental or biological factors directly trigger or amplify STX production. By combining controlled experiments, field data, and AI-driven predictive modeling, this integrative approach provides a comprehensive framework for understanding and forecasting saxitoxin dynamics in a changing and biologically interactive ocean.

## 6. Potential Impact and Ecological Implications

The integration of AI into saxitoxin research has the potential to be truly transformative, redefining decades of traditional studies and accelerating discovery in ways previously unattainable. By rapidly identifying latent toxigenic species and predicting harmful algal bloom (HAB) dynamics, AI can revolutionize monitoring, early warning systems, and risk assessment for human and ecosystem health [96]. On the molecular and evolutionary front, AI can illuminate the diversification, retention, and horizontal transfer of *sxt* genes across dinoflagellate lineages, resolving longstanding uncertainties about saxitoxin biosynthesis and regulatory mechanisms. Coupled with environmental and biological interaction data, predictive AI models can forecast toxin production under changing climate and nutrient conditions, enabling proactive ecosystem management and informed policy decisions.

Beyond immediate advances in monitoring and prediction, the integration of AI introduces a paradigm shift in how saxitoxin research is conceptualized and operationalized. AI-driven frameworks enable continuous learning from expanding global datasets, allowing models to evolve alongside changing ocean conditions and emerging toxin profiles. This adaptive capacity supports the translation of fundamental molecular insights into real-world applications, including dynamic risk mapping, decision-support tools for fisheries and public health agencies, and the prioritization of surveillance efforts in data-limited regions. By unifying molecular processes with large-scale environmental variability, AI establishes a scalable research infrastructure that bridges discovery science and applied management, positioning saxitoxin research within a broader system of global ocean intelligence and long-term resilience planning [1].

## 7. Current Limitations, Challenges, and Future Perspectives

Despite its transformative potential, the application of AI to saxitoxin research faces several major limitations [30,135]. One of the most fundamental is the lack of accurate identification of *sxt* genes in dinoflagellates, which remains extremely challenging due to fragmented transcripts, extensive paralog expansion, and limited biochemical validation. However, AI frameworks, such as DeepBGC for biosynthetic gene cluster detection, DeepARG for fragmented gene classification, and protein language model–based tools like ESM-2, ProtT5, and DeepFRI, demonstrate that deep learning can reliably detect complex or divergent biosynthetic genes in other organisms [104,106,120]. Building on these approaches, we propose a dedicated AI-driven platform, available as a web or standalone tool, capable of predicting *sxt* genes from transcriptomic or genomic data with far higher accuracy than current methods. Leveraging deep-learning classifiers, protein language models, and embedding-based orthology inference, it would distinguish true biosynthetic genes from homologs or partial fragments, assign orthologous and paralogous relationships, and provide confidence scores [104]. This scalable system would enable rapid, standardized annotation across species, integrate with evolutionary analyses, and reduce reliance on labor-intensive validation, accelerating saxitoxin research and improving reproducibility.

Furthermore, the genomic investigation in dinoflagellates remains constrained, due to the scarcity of high-quality, well-annotated multi-omics datasets for dinoflagellates [22], with most discoveries limited to a few characterized genes [18,100] with others identified through scattered transcriptomic approaches [39,43,52]. Aside from a few draft genomes within *Symbiodinium* [179,180], comprehensive genomic resources for other dinoflagellates, especially regarding the saxitoxin biosynthesis, are largely lacking. Overall, the extremely large, repetitive genomes, scattered transcriptomes, and unexplored proteomes and metabolomes restrict the ability of AI models to learn accurate biological or biochemical patterns [181]. In addition, these challenges are compounded by the inherent difficulty of sequencing dinoflagellates, slowing the development of reference-quality genomic resources needed for pathway and gene cluster inference. However, as omics technologies advance and more data enter public repositories, there is an opportunity to decode saxitoxin biosynthesis. Therefore, instead of relying on isolated omics studies, a fully integrated multi-omics framework across multiple species is needed [22]. Using unified extraction and processing pipelines will generate comparable transcriptomics, proteomics, metabolomics, and environmental datasets from the same material. Applying advanced AI and machine-learning tools to these combined datasets will reveal the regulatory architecture of saxitoxin biosynthesis and enable predictive modeling of species-specific pathways and environmental drivers of toxicity [135,140].

Harmful algal blooms (HABs) are inherently complex ecological phenomena shaped by interacting with biotic and abiotic factors, generating highly multidimensional datasets [182,183]. Yet many coastal regions still lack long-term, high-frequency monitoring programs, and existing datasets often contain substantial gaps, such as missing information on nutrients, hydrodynamics, pollutants, or microbiome structure. Although new sensing technologies and data platforms are emerging, they frequently lack the historical depth required for reliable HAB forecasting [184]. These limitations create severe data imbalance and restrict model generalizability, causing AI systems to perform well in one region or species but fail when applied to different ecosystems, taxa, or climate-driven scenarios. Importantly, such limitations directly affect the reliability of food safety risk assessments, the prediction of paralytic shellfish poisoning (PSP) events, and the timely implementation of aquaculture harvesting restrictions and area closures.

However, despite the complexity of HABs and persistent gaps in biological and toxin-specific datasets, the rapid expansion of open-access physical and chemical ocean databases offers a path forward [16]. Global platforms such as NASA OceanColor (MODIS/VIIRS), NOAA ERDDAP, and the World Ocean Database now provide high-resolution, long-term data on temperature, chlorophyll, nutrients, and circulation that can be systematically integrated with biological, molecular, and toxin-profile datasets [185,186,187]. Future HAB research will increasingly rely on AI frameworks capable of fusing these heterogeneous datasets, handling data imbalance, and transferring knowledge across regions and species. Such integrative approaches will enhance model generalizability, facilitate region-independent forecasting, and support early warning systems for toxin-producing blooms under ongoing climate change. Ultimately, these advances will strengthen food safety surveillance, enhance HAB prediction under climate change, and provide actionable decision-support tools for marine resource management and aquaculture risk prevention.

Importantly, a lot of these issues are related to financial and resource constraints. Sustained financial investment has been repeatedly identified as a critical prerequisite for effective HAB monitoring, multi-omics integration, and AI-driven forecasting, particularly for maintaining long-term datasets and computational infrastructure [23,28,181]. High-performance computing, sequencing budgets, specialized monitoring programs, and steady funding streams are all unavailable to many research groups where HABs are growing quickly [16]. Furthermore, the process of creating genomic data is time-consuming and labor-intensive by nature, and it is likely that the goals and resources of the staff members involved in their collection do not include the use of ML and AI for HAB modeling [188,189]. This leads to disparities in the ability to use AI and create predictive STX frameworks. Addressing current financial and resource constraints will be critical for advancing AI-driven HAB and saxitoxin research. Future progress will likely depend on coordinated international funding, shared infrastructure, and open-access multi-omics and environmental databases that reduce duplication of effort and lower entry barriers for under-resourced regions. Advances in cost-efficient sequencing, cloud-based high-performance computing, and automated AI workflows are expected to make large-scale analyses more accessible.

AI models, particularly deep learning architectures, can face several challenges in multi-omics studies [189]. High-dimensional data with limited samples increases the risk of overfitting, reducing the model’s ability to generalize to new datasets [190]. Small sample sizes, common in dinoflagellate studies, further exacerbate overfitting and can produce spurious or unstable predictions [191]. Additionally, many AI models act as “black boxes,” limiting interpretability and making it difficult to extract biologically meaningful insights [192]. These issues can be mitigated through careful feature selection, regularization, cross-validation, data augmentation, transfer learning, and the use of explainable AI techniques [193]. However, efficient application of these technologies demands interdisciplinary partnerships. Therefore, establishing strong collaborations among biologists, data scientists, and AI/ML specialists is essential to address the unique challenges at the interface of AI and multi-omics in dinoflagellate toxins

Finally, barriers related to expertise, standardized workflows, computational access, and data-sharing policies further slow progress. Overall, overcoming these challenges will require sustained funding, international collaboration, and coordinated efforts to expand datasets, improve interpretability, and ensure that AI tools capture the biological and ecological realities of saxitoxin production to fully realize the potential of AI-driven research in unraveling decades of unknowns of the most potent marine toxins amid a changing climate.

## 8. Conclusions

As climate change increases, the intensity of HABs is expected to rise, increasing the risk of PSP caused by dinoflagellate saxitoxin, with profound impacts on socio-economic systems, ecosystem health, and human well-being [4,15]. In this review, we summarized the current knowledge on dinoflagellate STX biosynthesis and highlighted persistent knowledge gaps, such as fragmented genomic data, unknown gene origins, uncertain annotations, unresolved biosynthetic steps, and inconsistent environmental observations, that have slowed progress toward an understanding of STX evolution, biosynthesis, and ecological dynamics. In this perspective, we suggest the application of AI and machine learning to overcome these limitations, offering a critical advancement for saxitoxin research and HAB forecasting. By integrating advanced AI tools with experimental biology, researchers can establish a comprehensive, scalable, and predictive framework to understand and anticipate saxitoxin production in a rapidly changing ocean, marking a transition from decades of inconsistent findings to a new era of comprehensive insight, where AI bridges the gap between data, mechanism, and actionable knowledge.

## Figures and Tables

**Table 1:** Summary of inconsistent findings and major knowledge gaps in dinoflagellate saxitoxin (STX) research.

Species	Major Outcome	Reference
Incomplete or Fragmented *sxt* Gene Clusters (Genomic Uncertainty)		
*Alexandrium* spp.	Phylogeny shows inconsistent clustering of *sxt* genes	[48]
*Alexandrium*	Genomic organization varies across strains	[67,72]
*A. fundyense*	Partial *sxt* genes; cluster not assembled	[34]
*A. tamarense*	Many isoforms; unclear genomic arrangement	[36,50]
*A. minutum*	*sxtA* copy number varies; there is no complete cluster	[35,57]
*A. minutum*	Fragmented *sxt* genes across scaffolds	[37]
*Alexandrium*	Genomic fragmentation prevents cluster definition	[48]
*G. catenatum*	*sxtB* not detected in the transcriptome	[39]
*C. punctatum*	Possibly *sxtA* not present	[47]
Regulatory Complexity (Transcription, Splicing, Epigenetics Still Unresolved)		
*Alexandrium* spp.	Correlation between the presence of *sxtA4* and PST biosynthesis	[57,73]
*Alexandrium* spp.	*sxtA4* expression is inconsistent between toxic and nontoxic	[37,74,75]
Multiple	*sxt* genes are found in both toxic and nontoxic species	[37,50,55]
*Alexandrium*	No universal regulatory signature	[17]
*A. minutum*	P-limitation increases STX sometimes	[76]
*Alexandrium* spp.	Temperature affects *sxt* genes but inconsistently	[17,77]
*A. minutum*	N-source changes expression unpredictably	[78]
*G. catenatum*	Nutrients affect toxin profiles inconsistently	[79]
*G. catenatum*	Different N:P ratios did not alter PST content or toxin profiles	[80]
*Alexandrium* spp.	Multiple stressors produce nonlinear effects	[17,81]
*A. pacificum*	Metabolic inhibitors change saxitoxin biosynthesis	[64]
*P. bahamense*	Toxin production increases as cell abundance decreases	[82]
Functional Validation Limitations (Lack of Genetic Tools)		
Dinoflagellates	Gene knockdown is partial and inconsistent	[83]
Dinoflagellates	Protein function inference is limited	[69]
*Alexandrium*	Cannot confirm enzyme functions experimentally	[64]
*Alexandrium*	*sxt* gene diversity prevents functional inference	[84]
*A. fundyense*	High redundancy in transcripts	[34]
Dinoflagellates	Extreme gene copy numbers complicate validation	[85,86]
*A. tamarense*	Homologs too divergent to infer function	[50]
Environmental Modulation of Toxin Production (Highly Inconsistent)		
*A. fundyense*	P-limitation sometimes increases toxins	[87]
*A. fundyense*	P-effects depend on N co-limitation	[88]
*A. minutum*	N-source inconsistent with toxin production	[78]
*A. pacificum*	Salinity inconsistent	[19]
*P. bahamense*	Seasonal correlations weak	[89]
*P. bahamense*	Co-occurrence of *sxtA4*+ and *sxtA4*− genotypes	[90]
*Alexandrium* spp.	Temperature and salinity are strain-specific in toxin production	[60,62]
*G. catenatum*	Marine heatwaves variable	[91]
*A. affine*	Temp/nutrient effects are unpredictable	[92]
*A. catanella*	Low temperature increases the toxin production	[21]
*A. pacificum*	Nitrate concentration influenced STXs production	[18]
*A. catanella*	Varying iron concentration altered growth and toxin production	[93]
*G. catenatum*	Salinity, nutrients, and temperature affect toxin contents	[60]
*Alexandrium* spp.	Temperature alters *sxt* genes inconsistently	[77]
*Alexandrium*	Combined climate change stressors are nonlinear	[81]
*A. catenella*	Cyst-forming bacteria influence toxin production	[66]
*A. minutum*	Light and N interactions are unpredictable	[78]
Evolutionary Origins of *sxt* Genes (Still Not Resolved)		
*Alexandrium*	*sxtA* evolution unclear	[36,50]
Dinoflagellate	*sxtA*, *sxtG*, and *sxtB* originated from cyanobacteria via HGT	[36]
*A. tamarense*	Divergent homologs complicate ancestry	[36]
Dinoflagellates	Acquire *sxt* genes independently from cyanobacteria	[34]
Dinoflagellates	Multiple independent acquisitions and losses of *sxt* genes	[50]
*Alexandrium*	Evolutionary relationships unstable	[67]
*Alexandrium*	Polyphyletic origins of *sxt* genes	[42]
*A. fundyense*	Polyploidy obscures origins	[34]
*G. catenatum*	*sxt* genes have polyphyletic origins, distinct from *Alexandrium*	[39,51]

## Data Availability

No new data were created or analyzed in this study.

## Abbreviations

The following abbreviations are used in this manuscript:

UN	United Nations
STX	Saxitoxin
STXs	Saxitoxin and its analogs (STX, GTX, C1, dSTX, etc.)
PSP	Paralytic Shellfish Poisoning
HABs	Harmful Algal Blooms
AI	Artificial Intelligence
ML	Machine Learning
*sxt*	Saxitoxin biosynthesis gene(s)
HGT	Horizontal Gene Transfer
CNN	Convolutional Neural Network
PKS	Polyketide Synthase
FAS	Fatty Acid Synthase
ESM-2	Evolutionary Scale Modeling version 2
ProtT5	Protein T5
ProtBERT	Protein BERT
DeepFRI	Deep Functional Residue Identification
GNN	Graph Neural Network
VAEs	Variational Autoencoders
iGRN	Integrative Gene Regulatory Network
LC–MS/MS	Liquid Chromatography–Tandem Mass Spectrometry
Y2H	Yeast Two-Hybrid
co-IP	Co-Immunoprecipitation
AP-MS	Affinity Purification Mass Spectrometry
DeepMET	Deep Learning Metabolomics Framework
DeepMass	Deep Learning Mass Spectrometry Framework
MOFA+	Multi-Omics Factor Analysis Plus
DRS	Direct RNA Sequencing
LSTM	Long Short-Term Memory
GRU	Gated Recurrent Unit
NASA	National Aeronautics and Space Administration
MODIS	Moderate Resolution Imaging Spectroradiometer
VIIRS	Visible Infrared Imaging Radiometer Suite
NOAA	National Oceanic and Atmospheric Administration
ERDDAP	Environmental Research Division’s Data Access Program
WOD	World Ocean Database
PST	Paralytic Shellfish Toxins
N:P	Nitrogen-Phosphorous ratio
ESM	Evolutionary Scale Modeling
ProtTrans	Protein Transformers
RNN	RNN
XGBoost	XGBoost
HDBSCAN	Hierarchical Density-Based Spatial Clustering of Applications with Noise
SHAP	SHapley Additive exPlanations
ESMFold	Evolutionary Scale Modeling Fold
SVM	Support Vector Machine
RF	Random Forest
MS2CNN	Mass Spectrometry to Convolutional Neural Network
WGCNA	Weighted Gene Co-expression Network Analysis
GG Models	Gaussian Graphical Models
ConvLSTM	Convolutional Long Short-Term Memory
EL	Ensemble learning
RL	Reinforcement learning
TT	Temporal Transformers
